# Setting up strategies: patient inclusion in biobank and genomics research in Europe

**DOI:** 10.1186/1750-1172-9-S1-P2

**Published:** 2014-11-11

**Authors:** Pauline McCormack, Anna Kole

**Affiliations:** 1 Policy, Ethics & Life Sciences Research Centre (PEALS), Newcastle University, Newcastle upon Tyne, Tyne and Wear, NE1 7RU, UK; 2 EURORDIS Plateforme Maladies Rares, Paris, 75014, France

## 

Rare disease patient organisations have a tendency to be deeply involved in research development and infrastructures, and are practiced at founding strategic alliances with clinical and research networks [1]. In building an integrated platform for rare disease databases, registries, biobanks and bioinformatics through the RD Connect project, we wanted to explore explicitly and in detail, the inclusion of rare disease patients and their advocates in RD Connect’s core activities.

RD Connect collaborates closely with two related research projects EURenOmics (rare kidney disease) and Neuromics (rare neuromuscular/neurodegenerative disease) both of which utilise genomic technologies to improve care and therapies for specific disease groups. A workshop of 45 clinicians, scientists and patients/advocates from RD Connect, EURenOmics and Neuromics, identified two areas of concern. The first were procedural, around the inclusion of patients in governance including: on-going dialogue between researchers and patients; mutual education; and reporting and dissemination. The second set were contemporary ethical, legal and social issues (ELSI), around privacy, informed consent, data sharing, return of results and incidental findings. A review of the literature found that rare disease patients’ views on these contemporary issues are rarely documented.

To explore these two areas we have parallel initiatives designed to include patients through membership of boards and committees at the highest levels and via a specific research strand investigating ELSI. Through including patients in governance, RD Connect fulfils the top level of Arnstein’s ladder of participation, that of citizen control, whereby those who the governance structure serves are represented in decision making [2]. Among other things, a Patient Advisory Committee works to discuss and build consensus on issues affecting patients and a Patient and Ethics Council promotes dialogue on ELSI between patients/advocates and researchers within RD Connect, Neuromics and EURenOmics.

In our research strand we are exploring patient hopes, expectations, concerns and fears for the creation of an integrated platform for rare disease databases, registries and biobanks in a series of focus groups and a Delphi exercise. Using the notion of communities of practice, which encourage collective learning through shared endeavour, we aim to use the findings from this research to inform discussion and education activities which will enable patient perspectives to be embedded into the work of RD Connect, EURenOmics and Neuromics [3]. Bearing in mind that patient organisations and scientific groups may have different decision making mechanisms, we will experiment with creating spaces to allow meaningful, on-going dialogue between patients/advocates and researchers [4].
